# Genetic diversity and volatile oil components variation in *Achillea fragrantissima* wild accessions and their regenerated genotypes

**DOI:** 10.1186/s43141-021-00267-3

**Published:** 2021-10-25

**Authors:** Abdelfattah Badr, Hanaa H. El-Shazly, Mahmoud Sakr, Mai M. Farid, Marwa Hamouda, Eman Elkhateeb, Hanan Syed Ahmad

**Affiliations:** 1 Botany and Microbiology Department, Faculty of Science, Helwan University, Cairo, 117900 Egypt; 2 Department of Biological Sciences and Geology, Faculty of Education, Ain Shams University, Cairo, 11341 Egypt; 3 Department of Plant Biotechnology, National Research Center, Cairo, Egypt; 4 Department of Phytochemistry and Plant Systematics, National Research Centre, Cairo, Egypt; 5 Botany and Microbiology Department, Faculty of Science, Tanta University, Tanta, 31527 Egypt

**Keywords:** *Achillea fragrantissima*, Essential oil, Genetic diversity, In vitro regeneration

## Abstract

**Background:**

Wild medicinal plants are suffering natural environmental stresses and habitat destruction. The genetic diversity evaluation of wild accessions and their in vitro raised genotypes using molecular markers, as well as the estimation of substances of pharmaceutical value in wild plants and their regenerated genotypes are convenient approaches to test the genetic fidelity of regenerated plants as a source of substances of pharmaceutical value. In this study, the genetic diversity of 12 accessions of the medicinal plant *Achillea fragrantissima*, representing five sites in the mountains of South Sinai, Egypt, were estimated by the inter simple sequence repeats (ISSR) fingerprinting and their volatile oil components were identified using gas chromatography-mass spectrometry (GC-MS) analysis. The same accessions were regenerated in vitro and the genetic diversity and volatile oil components of propagated genotypes were determined and compared to their wild parents.

**Results:**

Clustering and principal component analyses indicated that the wild accessions and their regenerated genotypes were genetically differentiated, but the regenerated plants are relatively more diverse compared to their wild parents. However, genetic variation between wild accessions is inherited to their in vitro propagated genotypes indicating genotypic differentiation of the examined accessions. The number of volatile oil compounds in the wild *A. fragrantissima* accessions was 31 compounds while in the in vitro propagated plants only 24 compounds were detected. Four major compounds are common to both wild and regenerated plants; these are artemisia ketone, alpha-thujone, dodecane, and piperitone.

**Conclusions:**

Genome profiling and essential oil components analysis showed variations in *A. fragrantissima* accessions from different populations. Genetic differences between wild and regenerated genotypes were analyzed and validated with the final conclusion that in vitro conditions elicited higher genetic variation that is associated with reduced amount and diversity in the essential oil components.

**Supplementary Information:**

The online version contains supplementary material available at 10.1186/s43141-021-00267-3.

## Background

Most medicinal plants, particularly in the arid environments, are decreasing in the wild due to weak regeneration under natural environmental stresses such as drought and salinity, and habitat destruction due to heavy overuse as well as overgrazing, uncontrolled collection, mining , and urbanization [1–3]. Therefore, conservation of wild medicinal plants using in vitro propagation is a vital approach to preserve their medicinal and pharmaceutical value. For this objective, it is important to evaluate the genetic variability in natural populations and also preserve genetic variability in the in vitro raised genotypes. The genetic diversity of wild and in vitro raised plants has been evaluated using genome profiling by using different molecular markers [3–5]. The genome profiling of plants with pharmaceutical value, using different molecular fingerprinting methods, was reviewed by Gantait et al. [6]. Several of these methods have been applied to address the genetic fidelity of in vitro propagated plants, examples include *Hedychium coronarium* by inter simple sequence repeats (ISSR) [7], the endangered medicinal plant *Rauwolfia tetraphylla*, by start codon targeted polymorphism (SCoT), ISSR, and random amplified polymorphic DNA (RAPD) [8], *Helianthus verticillatus* in vitro raised plants using SSRs [9] and the blackberry (*Rubus fruticosus* L.) using RAPD and sequence-related amplified polymorphism (SRAP) [10].

Plant regeneration by in vitro systems is generally associated with the presence of variations among regenerants [11, 12]. These variations may be connected to epigenetic modifications occurring in an individual’s genome that ultimately influence the organism’s development and the process of its heredity [13, 14]. In this connection, Zarreen et al. [15] reported 33% and 25% molecular polymorphism between wild type and in vitro regenerated *Aloe Vera* plantlets using RAPD and ISSR markers analysis respectively. The ISSRs and SSAP markers were able to detect soma-clonal variation in the callus of *Artemisia absinthium*, while no variation was detected in the plants regenerated from the nodal explants [16]. Few reports are available on the preservation of the chemical substances that have pharmaceutical value and the genetic diversity of regenerated genotypes. In *Silybum marianum*, growing in different geographic locations in Iran, RAPD markers were found associated with variation in the content of the major pharmaceutical substance, silymarin [17], and *Thymus vulgaris* clones were distinguished as five chemotypes using 12 ISSR primers [18].

*A. fragrantissima* is a very well-known medicinal plant [19]. It is a desert plant used in traditional medicine in the Arabian region for the treatment of various illnesses such as hepatobiliary disorders, skin inflammations, inflammatory disorders, hypoglycemia, diabetes lowering blood cholesterol levels, and as an appetite enhancing drug [19, 20]. The essential oil extracted from *A. fragrantissima* exerts a bactericidal effect on *Escherichia coli* cells [21]; *Candida albicans* [22] and its extracts have antiviral activity against polio [23]. Phytochemicals present in the *A. fragrantissima* extract could be beneficial in preventing or treating neurodegenerative diseases [24] and for cancer prevention activity in the treatment of chronic myeloid leukemia cells [25]. The diversity among the populations of *A. fragrantissima* in Egypt based on morphological variation and ISSR marker profiling was previously evaluated by Badr et al. [26] and the genetic diversity and population structure of *A. fragrantissima* in South Sinai in Egypt was recently reported [27] and the essential oil components have been investigated in connection with their antimicrobial activity [28, 29] and components of aerial parts of plants from Egypt and Saudi Arabia were recently compared [30].

The objectives of the present study are to address genetic diversity as measured by ISSR fingerprinting polymorphism and variation in the chemical composition in *A. fragrantissima* wild accessions and their regenerated genotypes and to report for the first time the genetic fidelity of the in vitro regenerated plants and the correlation between the genetic diversity and chemical variation in wild plants and *in-vitro* derived plants.

## Methods

### Plant collection and seed germination

Twelve accessions of *A. fragrantissima* were collected, as individual plants were selected for the current study, from five sites in the mountains of South Sinai. The area, site, GPS location, number of samples, and elevation of localities from which the examined accessions are collected are given in Table 1. For each accession, ten key morphological traits were measured, and the values of their measurements are given in Table 2.

**Table 1:** The area, site, GPS location, number of samples and elevation of localities from which the examined *A. fragrantissima* accessions were collected

Area	Population code	GPS location	Elevation	Sample Code	Germination %	
					Paper	MS medium
Wadi El-Arbaein St. Catherine	W 1	N: 28.55399° E: 033.9497°	1583 (m)	W 1-1	64%	78%
				W 1-2	58%	75%
				W 1-3	52%	70%
				W 1-4	56%	73%
				W 1-5	42%	65%
South Sinai, east of Saint-Catherine	W 2	N: 28.69700° E: 034.0408°	1348 (m)	W 2-1	57%	77%
				W 2-2	74%	80%
				W 2-3	55%	71%
South Sinai, west of Saint-Catherine	W 3	N: 28.64873° E: 033.89046°	1104 (m)	W 3-1	64%	66%
				W 3-2	68%	70%
	W 4	N: 28.69000° E: 033.98115°	1262 (m)	W 4-1	68%	70%
	W 5	N: 28.77283° E: 034.17673°	1072 (m)	W 5-1	58%	75%

**Table 2:** Measurements of ten morphological traits for 12 samples of *A. fragrantissima*

Plant code	Plant height	Crown diameter	Size index	Height/diameter	Leaf length (cm)	Leaf width (cm)	Head length (cm)	Head width (cm	Bracts length (mm)	Number of florets/dead
W 1-1	97	88	92.5	1.10	0.83	0.26	0.72	0.40	3.35	29
W 1-2	86	82	84.0	1.05	1.10	0.30	0.72	0.35	3.28	26
W 1-3	96	75	85.5	1.28	0.68	0.28	0.68	0.38	3.50	31
W 1-4	94	80	87.0	1.18	0.78	0.2	0.57	0.3	3.40	35
W 1-5	98	87	92.5	1.13	0.69	0.2	0.55	0.3	3.20	32
W 2-1	110	77	93.5	1.43	1.26	0.36	0.73	0.36	3.50	31
W 2-2	106	75	90.5	1.41	0.85	0.34	0.65	0.30	3.70	30
W 2-3	99	83	91.0	1.19	1.00	0.40	0.70	0.40	3.10	28
W 3-1	85	72	78.5	1.18	0.65	0.32	0.57	0.33	3.0	27
W 3-2	89	76	82.5	1.17	0.62	0.29	0.60	0.40	3.0	29
W 4-1	87	97	92.0	1.13	0.74	0.31	0.5	0.37	3.32	33
W 5-1	97	97	97.0	1.0	1.16	0.36	0.73	0.36	3.50	33

### In vitroregeneration of *A. fragrantissima*

Seeds of all accessions were surface sterilized by immersion in 70% ethanol for 60 s, and then rinsed in 20% NaOCl solution and drops of tween 20 for 20 min. Seeds were then washed three times with sterile distilled water and germinated on moist filter papers and basic MS mediums [31]. The percentage of germination on MS medium was much higher for all accessions (Table 1) and consequently, seedlings grown on MS medium were used for regeneration. For the initiation of regeneration, shoot explants of 1-month-old seedlings were cultured on full strength solidified basal MS medium supplemented with (0.5 mg/l BA + 0.5 mg/l NAA), which was the best media giving the highest number of shoots for most accessions after 1 month of incubation. Regenerated plants were acclimatized on soil containing Patmos and perlite in a ratio of 1:2 in small pots wrapped in a plastic bag and incubated under a photoperiod of 16/8 h light/ dark (Ca. 50 μ mol m^−2^ s^−1^) at a constant temperature of 25±1°C for 2 months and then transferred to 15 cm diameter pot for acclimatization.

### Methods of essential oil analysis

Fresh shoot parts of mature wild *A. fragrantissima* accessions were air-dried in shade at room temperature until constant weight. The dried material was grounded with a blender to a fine powder and stored at 4°C in a tightly covered bottle. For regenerated genotypes, vegetative parts of acclimatized plants were also dried at room temperature to a constant weight, powdered in a blender, and stored at 4°C in a tightly covered bottle. For essential oil extraction, 100 gm of the powdered material was distilled in a modified Clevenger apparatus using distilled water for approximately 3 h. The oil prepared in each case passed over anhydrous sodium sulfate to be dried and was stored at − 20°C until further analysis. The extracted oil was dehydrated over anhydrous sodium sulfate and kept in the refrigerator until used for the gas chromatography-mass spectrometry (GC-MS) analysis. The GC-MS analysis was carried out at the Department of Medicinal and Aromatic Plants Research, National Research Center with the following specification using a TRACE GC Ultra Gas Chromatographs (THERMO Scientific Corp., USA), coupled with a THERMO mass spectrometer detector (ISQ Single Quadrupole Mass Spectrometer). The GC-MS system was equipped with a TR-5 MS column (30 m × 0.30 mm i.d., 0.25 μm film thickness). Analyses were carried out using helium as carrier gas at a flow rate of 1.0 mL/min and a split ratio of 1:10 using the following temperature program: 60°C for 1 min; rising at 3.0°C/min to 240°C and held for 1 min. The injector and detector were held at 300°C. Diluted samples (1:10 hexane, v/v) of 0.2 μL of the mixtures were always injected. Mass spectra were obtained by electron ionization (EI) at 70 eV, using a spectral range of *m/z* 40–450.

### ISSR fingerprinting

DNA extraction and PCR procedures for the ISSR fingerprinting were performed as described by Badr et al. [26] using the properties and sequence of the primer listed in Table 3. The ISSR markers for wild accessions and their regenerated plants were visualized and the size of each marker was estimated and scored as either present or absent in a data matrix and used for calculating the genetic diversity and also for calculating the correlation between ISSR polymorphism and variation in essential oil components in wild accessions and regenerated plants.

**Table 3:** Primers codes, sequences (Y = C or T) as well as the total number of amplified alleles (total), polymorphic alleles (polym), monomorphic alleles (mono) unique alleles (unique) and % of total polymorphism induced in wild and regenerated accessions of *A. fragrantissima*

Primer	Sequence (5-3)	Number of amplified alleles in wild accessions				Number of amplified alleles in regenerated accessions			
		Total	Polym	Mono	Unique	Total	Polym	Mono	Unique
807	(AG)8 T	5	3	1	1	6	5	1	0
809	(AG)8 G	5	4	1	0	6	3	2	1
814	(CT)8 TC	5	3	1	1	7	6	1	0
825	(AC)8 T	4	2	1	1	5	4	1	0
834	(AC)8 YT	6	5	1	0	6	5	1	0
841	(GA)8 YC	5	4	1	0	6	5	1	0
844-B	(CT)8 GC	6	4	1	1	6	3	2	1
17898-B	(CA)6 GT	9	7	1	1	9	5	3	1
17899-A	(CA)6 AC	5	4	1	0	5	5	0	0
17899-B	(CA)6 GG	5	3	2	0	7	5	2	0
HB-08	(GA)6 GG	5	4	1	0	7	5	2	0
HB-09	(GT)6 GG	8	6	1	1	8	6	2	0
HB-10	(GA)6 CG	7	6	1	0	7	5	2	0
HB-11	(GT)6 CG	7	6	1	0	7	5	2	0
HB-12	(CAC)3 GC	8	7	1	0	8	6	2	0
HB-13	(GAG)3 GC	7	5	1	1	7	4	2	1
HB-14	(CTC)3 GC	6	5	1	0	6	4	2	0
HB-15	(GTG)3 GC	5	4	1	0	5	4	1	0
UBC-820	(GT)8 C	6	5	1	0				
UBC-827	(AC)8 G	4	3	1	0				
**Total**		**118**	**90**	**21**	**7**	**118**	**85**	**29**	**4**
**Percentage**			**76.27**	**17.8**	**5.93**		**72.03**	**24.58**	**3.6**

### Data analyses

The number of ISSR amplified alleles including, polymorphic alleles, unique alleles, and the percentage of polymorphism revealed by each primer, in the examined wild and regenerated plants of the examined sites are given in Table 3. In scoring ISSR fingerprinting, polymorphic markers refer to alleles found in at least two samples, and a unique allele is found in one sample whereas monomorphic markers are found in all samples, the latter markers did not differentiate samples from each other.

Genetic diversity among the 12 individual wild accessions and their regenerated genotypes was calculated based on the binary data from the amplified ISSRs alleles using the Community Analysis Package-5 (CAP) by Seaby and Henderson [32], and an average linkage tree based on hierarchical grouping function [33] was constructed. The PAST (Paleontological Statistics) software [34] was also used to construct a tree, based on the Euclidean distance using the Unweighted Pair Group Method with Arithmetic Mean (UPGMA). A scatter plot of the examined plant accessions was also made using the principal coordinates analysis (PCA) in the PAST software to illustrate the grouping of the 24 *A. fragrantissima* plant samples including the 12 wild accessions and the regenerated plants based on variation in the polymorphism in the ISSR alleles.

The pheatmap and ggplot2 packages [35, 36] were used to visualize the similarity of wild accessions and their in vitro regenerated genotypes based on the variation in volatile oil components. A correlation matrix was created using the proportions of components by MrBayes [37]. The “actoextra” package of Kassambara and Mundt [38] was also used to extract and visualize the results of multivariate data analyses to produce a heatmap. In the heatmap, the scale of color is relative to the value of the divergence between investigated values. Pearson correlation values between the morphological and the chemical composition for the 12 wild accessions of *A. fragrantissima* were calculated using two variables by the R-software package, and the correlation coefficients were expressed as complot [39]. Meanwhile, Pearson correlation coefficients correlation values between volatile oils constituents and the ISSR alleles for the wild accessions and the regenerated genotypes were calculated using the IBM SPSS Statistics Analysis Package.

## Results

### The ISSR fingerprinting

**Fig. 1:**
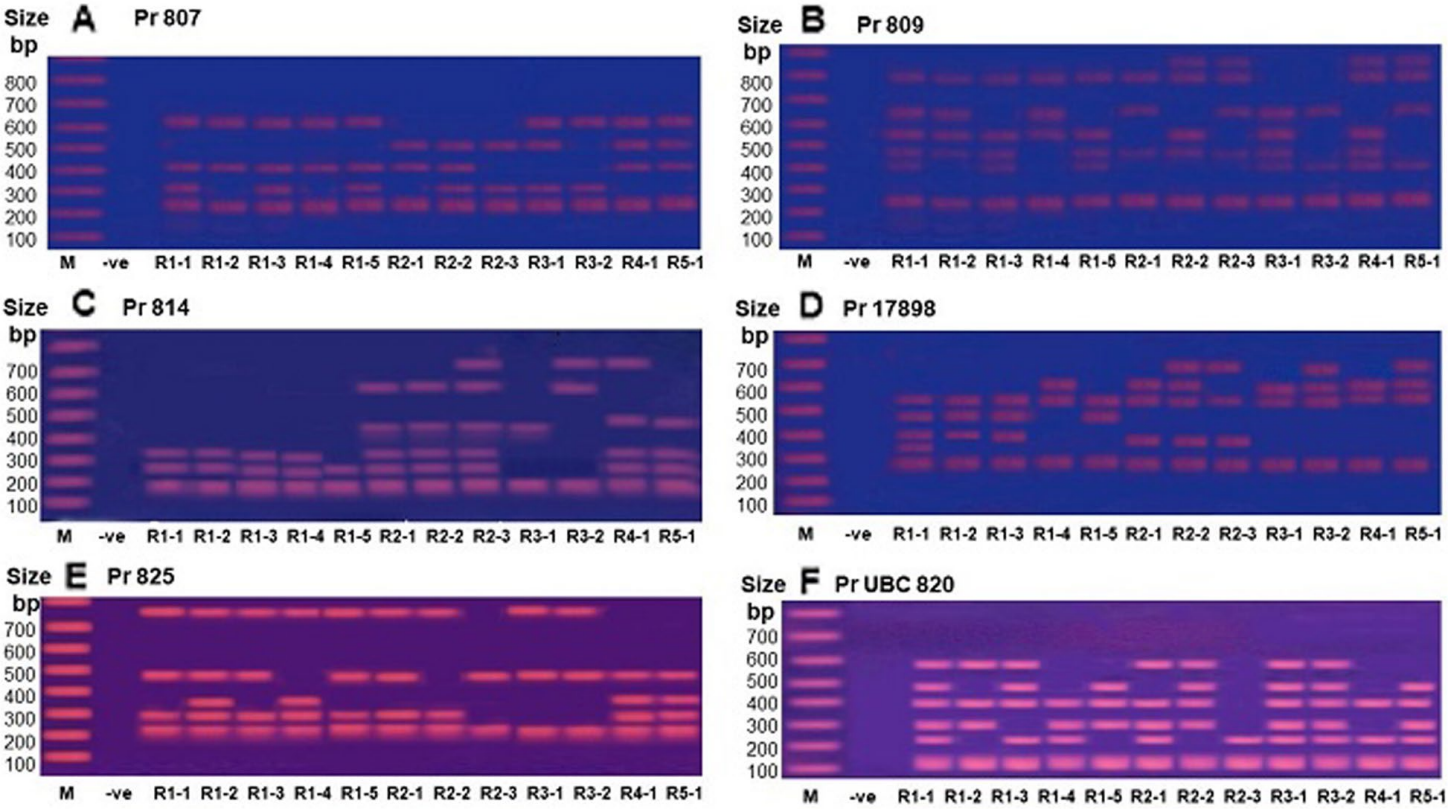
Photographs illustrating the ISSR fingerprinting of the 12 regenerated genotypes of *A. fragrantissima*, induced by the six primers. **A** pr 807, **B** pr 809, **C** pr 814, **D** pr. 17898, **E** pr 825, and **F** pr UPC 820

The results of the ISSR fingerprinting of the examined 12 wild individual accessions of *A. fragrantissima* from the five sites using 20 ISSR primers and their in vitro regenerated genotypes using 18 primers, are summarized in Table 3. A total of 118 ISSR bands (alleles) were amplified in all wild plants using the 20 primers and the same number was recorded in the regenerated plants using 18 primers. Figure 1 illustrates examples of ISSR fingerprints amplified in the 12 regenerated plants by the primers 807 (A), pr. 809 (B), pr. 814 (C), pr.17898 (D). pr. 825 (E) and pr. UBC 820 (F), respectively. A 100 bp ladder was used to indicate the molecular size of markers that ranged in size between 100 bp and 700 bp. The highest number of alleles (9 bands) were produced by 17898-B in wild plants and regenerated plants and the lowest number of alleles was produced by primer 825 in the wild plants. The percentage of polymorphism in wild plants is 76.27% and in the regenerated plants is 72.03. The number and percentage of monomorphic markers (alleles) are lower in wild plants compared to regenerated genotypes, whereas the number and percentage of unique alleles are higher in the regenerated plants compared to wild plants (Table 3).

### Genetic diversity among wild accessions and regenerated genotypes

**Fig. 2:**
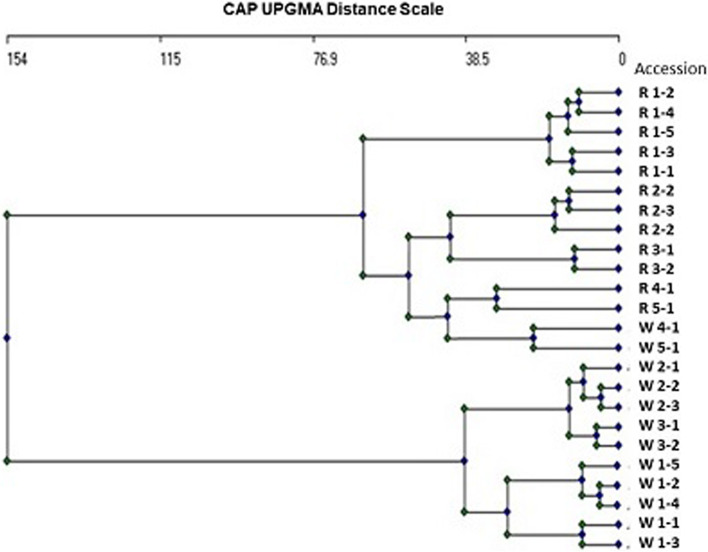
Genetic diversity among wild accessions and their regenerated genotypes expressed as a CAP average linkage tree based on hierarchical grouping function

**Fig. 3:**
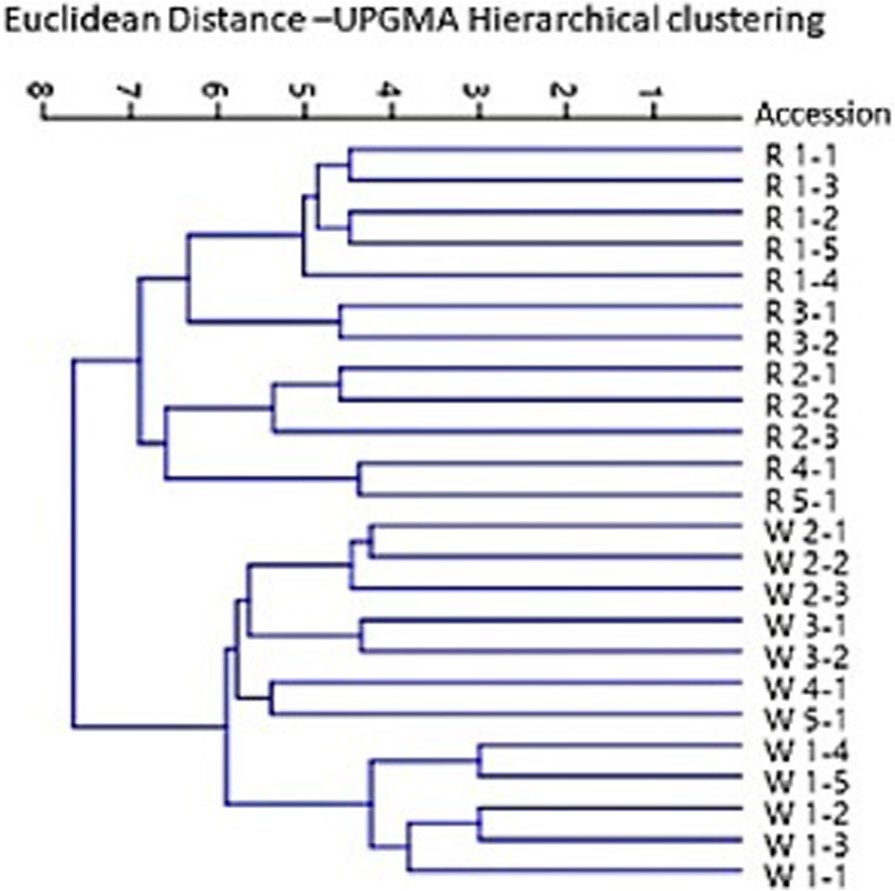
Euclidean UPGMA distance tree constructed using the PAST software differentiating all wild accessions from their regenerated plants

The genetic diversity among wild accessions and their regenerated plants expressed as a CAP average linkage tree based on hierarchical grouping function is illustrated in Fig. 2. Ten wild accessions representing site 1 (W 1-1, W 1-2, W 1-3 W 1-4, W 1-5), site 2 (W 2-1, W 2-2, W 2-3), and site 3 (W 3-1, W 3-2) are differentiated as a major group from the other two wild accessions from the two sites W 4-1 and W 5-1 and their regenerated plants which constitute another larger group of 14 samples. In the latter group, the two wild accessions W 4-1 and W 5-1 and their regenerated plants are grouped as a cluster comprising these four samples and the other two clusters, one comprising the five regenerated genotypes of site 1 (R 1-1, R 1-2, R 1-3 R 1-4, R 1-5), and the other comprises regenerated plants of the accessions of site 2 (R 2-1, R 2-2, R 2-3) and site 3 (R3-1, R 3-2). On the other hand, the Euclidean distance UPGMA tree constructed using the PAST software (Fig. 3); differentiated all wild accessions from their regenerated plants as two different groups. In each group, the five plants representing site 1, the three plants representing site 2, and the two plants of site 3 are distinguished as three clusters from the two samples representing site 4 and site 5 respectively.

**Fig. 4:**
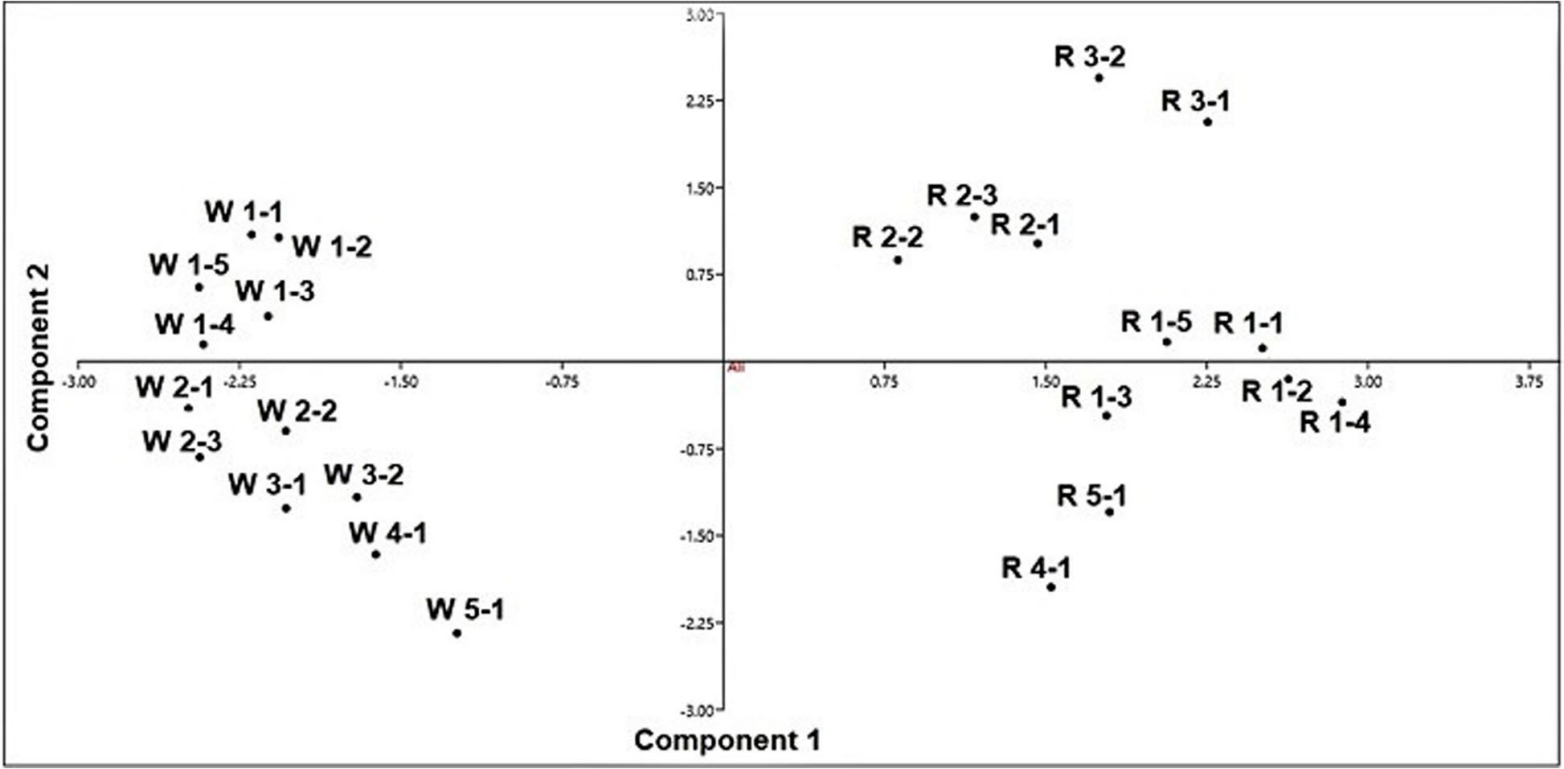
PCA scatter diagram illustrating the separation of the wild accessions from their regenerated genotypes, as two distinct groups and the differentiation of regenerated genotypes

The genetic differentiation of the wild accessions from their regenerated genotypes, based on the ISSR polymorphism is confirmed by a PCA scatter diagram, illustrating the grouping of the wild accessions and regenerated plants as two distinct groups (Fig. 4) which shows that regenerants from one population are closer to each other compared to accessions from different populations. In this diagram, the wild accessions are more closely related to each other whereas the regenerated genotypes are differentiated from each other. In addition, the genotypes regenerated from the same site are closer to each other compared to genotypes of different sites, this indicates that the genetic variation between wild accessions is inherited from their *in vitro* propagated genotypes.

### Essential oil constituents of wild and regenerated plants of *A. fragrantissima*

**Fig. 5:**
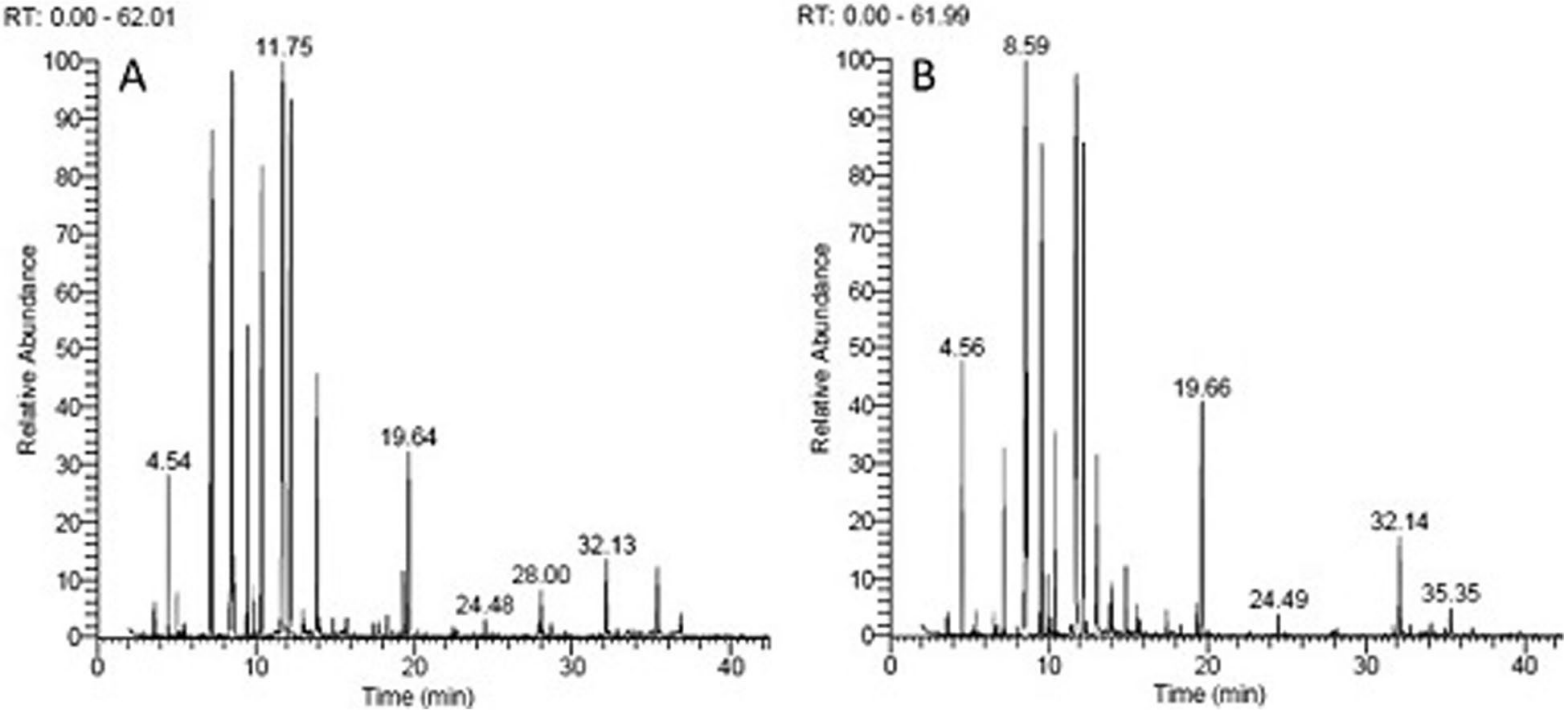
GC-MS total chromatogram for the volatile oil of the two *fragrantissima* wild accessions W1-3 and W 4-1

**Fig. 6:**
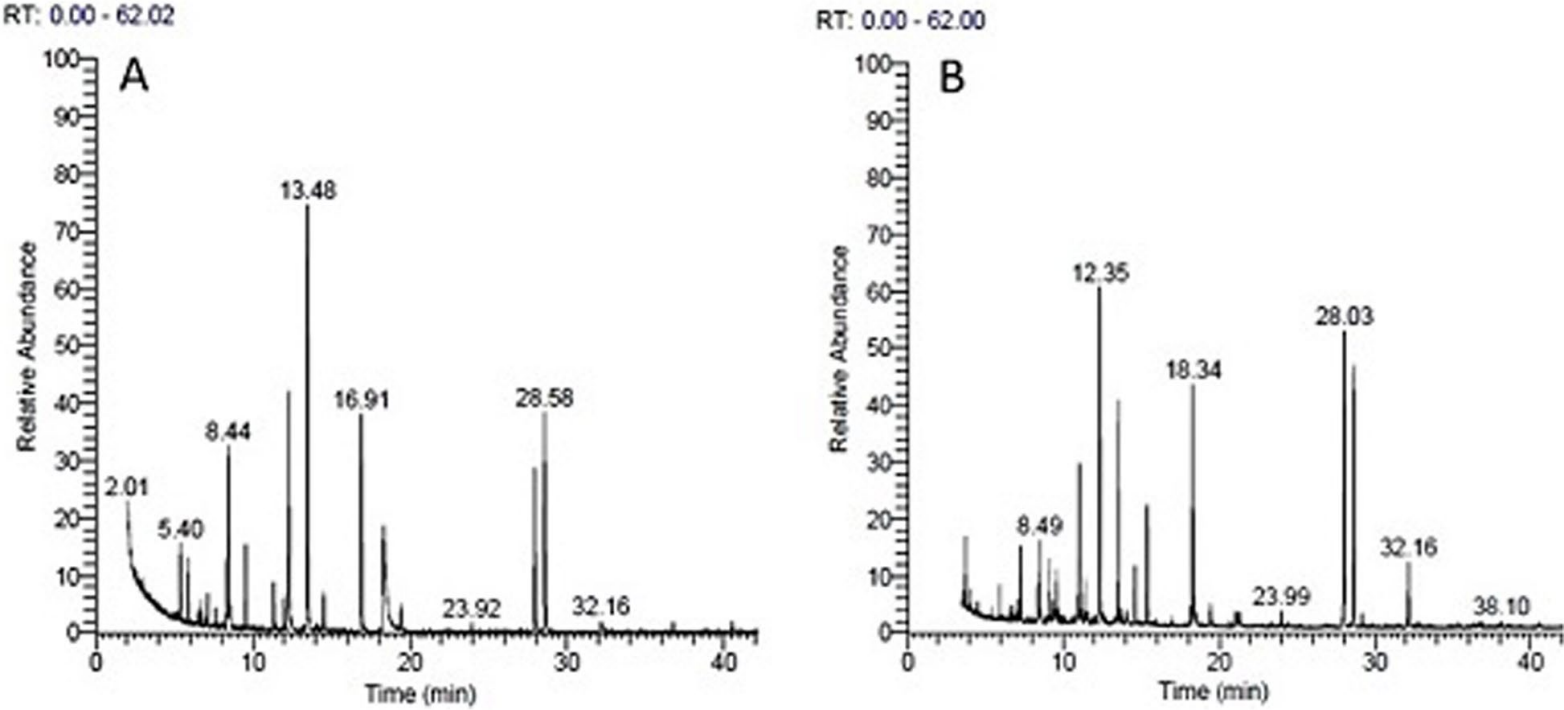
GC-MS total chromatogram for the volatile oil of the in vitro regenerated genotypes R1-3 and R 4-1 of the wild accessions W1-3 and W 4-1

The extracted liquid oil ranged in color from pale yellow oil to yellow with a strong penetrating pleasant odor. The amount of essential oil in the wild *A. fragrantissima* accessions was much higher compared to the regenerated plants (Tables 4 and 5). Its percentage in wild accessions ranges from 1.02% in the accession W3-1 to 2.18% in W1-3. However, it varies within accessions of sites 1, 2, and 3. In the regenerated genotypes, the amount of essential oil ranges from 0.28% in R3-1 to 0.68% in R4-1. The chemical constituents of the essential oil in *A. fragrantissima* analyzed by GC-MS resulted in the identification of 31 compounds in the oil of the wild accessions (Table 4) and 24 compounds in the regenerated plants (Table 5). Gas chromatography-mass spectrometry (GC-MS) total chromatogram for the volatile oil of two wild accessions and their regenerated genotypes are illustrated in Figs. 5 and 6, respectively. Four major compounds are common to both wild and regenerated plants; these are artemisia ketone, alpha-thujone, dodecane, and piperitone. The artemisia ketone, was a major compound in all accessions, in the wild accessions, it ranges between 17.6% in W1-1 and 10.5% in W4-1. In the regenerated genotypes, artemisia ketone was also a major compound in all genotypes; it ranged from 16.2% in RP1-3 to 9.11% in RP2-1. The piperitone amount ranges between 13.48% in W1-4 to 7.23% in W2-3 of the wild accessions. In the regenerated genotypes, piperitone ranged between 16.36% in RP5 to 8.48% in RP1-2. Meanwhile, alpha-thujone proportion ranged between 22.9% in W5.1 to 12.1% in W1.3. In the regenerated genotypes, the proportion of alpha-thujone ranged between 19.3% in R4.1 to 14.2% in R1.3. Dodecane was also a major compound in the wild and in vitro regenerated plants, but its proportion was higher in the regenerated genotypes, it ranged from 9.2% in W5.1 to 3.3% in W1.5 of the wild accessions (Table 4) and from 17.2% in RP3.2 to 8.6% in RP1.3 of the regenerated genotypes (Table 5).

**Table 4:** Retention time and components name and percentage of each component in the essential oil extracted from 12 individual plants representing five populations of *A. fragrantissima*

Ser	RT	Components name	W1-1	W1-2	W1-3	W1-4	W1-5	W2-1	W2-2	W2-3	W3-1	W3-2	W4-1	W5-1
		**Total essential oil %**	**1.22**	**1.24**	**2.18**	**1.56**	**1.65**	**1.33**	**1.13**	**1.30**	**1.02**	**1.51**	**2.04**	**1.45**
1	5.55	Santolina triene	3.35	3.02	3.95	2.89	2.91	3.9	3.57	3.24	3.13	2.52	1.38	1.62
2	6.56	Sabinene	0	0.8	0.23	0.45	0	0	0	0.33	0.23	0	0	0
3	7.20	Camphene	2.64	2.95	2.78	2.64	4.75	4.64	4.68	5.44	4.57	3.64	1.64	1.51
4	8.16	o-Cymene	0.19	1.4	0.26	0.77	0	0	0.39	0.55	0.46	0.51	0.69	0.42
5	8.66	p-Menthane-3,8-diol,cis-1,3,trans-1,4-	5.8	6.1	6.6	7.2	6.2	6.11	7.14	8.1	6.2	9.2	8.8	8.8
6	9.08	Santolina alcohol	14.6	13.2	14.2	10.5	17.1	11.3	10.6	10.7	8.1	6.62	8.22	11.3
7	9.65	Eucalyptol	0.66	1.14	0.89	1.56	1.45	1.43	1.6	1.39	1.02	0.96	7.86	0.23
8	10.60	Artemisia ketone	17.6	16	15.2	14.4	15	13.1	13.23	11.5	11.5	11.36	10.5	13
9	10.91	Hotrienol	0.82	0.78	0.85	0.78	0.69	0.81	0.83	1.3	0.79	0.64	0.77	1.07
10	11.15	Artemisia alcohol	4.27	4.26	4.75	3.01	3.87	4.43	3.8	3.72	4.76	4.6	5.72	4.42
11	11.72	β -thujone	1.8	2.27	3.26	3.01	3.67	2.85	3.6	3.73	4.76	4.74	0.44	0.42
12	12.18	alfa thujone	15.6	16.39	12.1	15.7	15.81	20.1	20.4	19.5	20.3	19.5	21.75	22.9
13	13.07	Isopinocarveol	0.57	2.6	0.4	0.03	0.41	0.82	0.54	0.67	0	1.84	2.41	2.86
14	13.54	Camphor	0.16	0.42	0.18	0.08	0.23	0.06	0.28	0.11	0.27	0.17	0,21	0.23
15	13.87	2-Methyl-2-octen-4-ol	2.93	2.04	2.08	3.22	1.78	1.77	1.86	0.5	5.57	6.5	3.23	3.29
16	14.01	(±) -Lavandulol	0.83	0.67	1.16	0.74	1.81	1.31	0.24	1.07	0.8	0.38	0.48	0.57
17	14.88	Terpinenene -4-ol	0.61	0.44	0.67	1.13	0.63	0.86	0.35	0.19	0.69	0.58	0.72	0.8
18	15.41	Dodecane	4.4	4.66	4.2	4.9	3.3	5.8	6.2	6.2	6.7	7.3	83	9.2
19	15.60	α-Terpineol	0.18	0.41	0.17	0	0	0	0	0.62	0.17	0.15	0.62	0
20	17.46	4α-ydroxyachipendol	0.29	0	0.34	0.46	0.24	0.59	0.28	0.51	0.26	0.34	0.27	0.52
21	19.35	Lavandulyl acetate	1.4	0.78	0.68	1.04	1.4	0.13	1.21	0.66	0.66	0.85	0.9	0.93
22	19.71	Sabinyl acetate	0.78	1.2	0.3	3.2	0.71	2.58	3.72	5.34	1.4	1.38	2.42	2.42
23	19.97	Piperitone	13.48	12.79	13.47	13.62	12.34	8.57	7.42	7.23	9.63	9.21	9.39	8.39
24	20.05	p-Cymen-7-ol	0	0.56	0	0.43	0	0	0	0	0	0	0.22	0
25	24.49	cis-Jasmone	0.44	0	0.13	0.43	0.54	0.55	0.34	0.46	0.26	0	0.24	0.27
26	27.88	Ethyl cinnamate	3.35	3.02	3.95	2.89	2.51	3.9	3.17	2.24	3.13	3.52	1.38	3.62
27	28.00	Germacrene-d	0	0.8	0.23	0.45	0	0	0	0.33	0.23	0	0	0
28	28.63	bicyclogermacrene	0.64	0.65	1.18	1.64	1.75	3.64	3.46	2.64	2.07	1.64	1.64	1.51
29	32.14	(+) spathulenol	0.59	0.4	0.26	0.77	0	0	0.39	0.55	0.46	0.51	0.29	0.42
30	34.08	Elemol	0.8	1.3	0.6	0.7	0.2	0.1	0.1	0.5	0.9	0.2	0.8	0.8
31	35.35	β-Eudesmol	0.66	1.14	0.89	1.16	0.45	0.43	0.6	0.39	1.02	0.96	0.86	0.23
		Total percentage	99.28	99.12	99.78	99.72	99.75	99.78	99.72	99.6	99.77	99.65	99.94	99.52

**Table 5:** Retention time and components name and percentage of each compound in the essential oil extracted from 12 regenerated plants representing 12 individual samples of six populations of *A. fragrantissima*

Ser	RT	Components name	R1-2	R1-2	R1-3	R1-4	R1-5	R2-1	R2-2	R2-3	R3-1	R3-2	R4-1	R5-1
		**Total essential oil %**	0.55	042	0.66	0.64	0.65	0.38	0.44	0.57	0. 28	0.53	0.68	0.48
1	4.55	Hexane	1.57	2.35	3.02	2.83	2.91	2.42	2.52	3.14	3.13	2.75	1.58	3.62
2	6.56	Sabinene	0	0.8	0.23	0.45	0	0	0	0.33	0.23	0	0	0
3	7.20	Limonene	13	13.8	15.2	16.9	15.2	12.6	11.3	13.27	14.8	17.7	7.73	6.44
4	8.36	o-Cymene	0.39	0.19	1.4	0.77	0	0.32	0.51	0.55	0.46	0.26	0.69	0.42
5	8.56	Camphene	3.3	2.78	2.88	2.79	2.21	1.86	1.93	1.36	2.77	0.75	2.21	1.8
6	8.65	Eucalyptol	0.6	0.66	4.14	1.56	0.45	3.09	5.96	6.39	1.02	0.89	1.86	0.23
7	9.91	Hotrienol	0.83	0.82	0.78	0.78	0.69	0.82	0.64	1.03	0.79	0.85	0.77	1.07
8	10.58	Artemisia ketone	16.2	14.6	17.0	16.4	10.02	9.11	9.3	10..0	13.5	14.2	14.5	15.0
9	11.18	Artemisia alcohol	4.8	4.27	2.26	5.01	3.87	2.85	3.6	3.7	2.76	3.75	4.72	4.42
10	12.16	alpha thujone	16	14.66	14.2	14.9	15.3	15.8	162	16.2	18.7	18.3	19.3	18.2
11	13.54	Camphor	2.86	3.93	1.04	2.22	3.78	4.32	1.5	0.5	1.57	2.08	3.23	3.29
12	14.01	(±)-Lavandulol	0.24	0.83	0.67	0.74	1.81	1.17	0.38	1.07	0.8	1.16	0	0
13	14.88	Terpinene-4-ol	0.35	0.61	1.44	1.13	0.63	1.14	0.58	1.39	0.69	0.67	0.72	0.8
14	15.41	Dodecane	10.2	10	8.6	9.9	11.3	12.8	13.3	13.2	14.3	17.2	10	10.2
15	15.60	α-Terpineol	0	0.18	0	0	0.41	0.4	0.15	0.62	0	0.17	0.62	0
16	19.35	1-Borneol	5.87	6.6	5.39	6.7	5.8	12.8	13.5	12.5	5.3	5.1	5.75	5.59
17	19.69	Sabinyl acetate	9.72	8.8	7.2	10.2	10.4	5.4	6.8	7.34	5.3	4.3	9.42	10.42
18	19.81	Piperitone	9.42	8.48	8.79	9..6	9.34	10.4	9.21	11.23	10.47	9.17	12.39	16.36
19	20.05	p-Cymen-7-ol	0	0.56	0	0.43	0	0	0	0	0	0	0.22	0
20	24.49	cis -Jasmone	0.34	0.44	0.56	0.83	0.54	0.19	0	0	0.26	0.13	0.04	0.27
21	27.88	Bicyclogermacrene	2.32	2.7	2.12	2.22	3.3	2.18	1.58	4.3	2.2	0.3	3.72	6.4
22	28.00	Germacrene-d	0	0.8	0.23	0.45	0	0	0	0.33	0.23	0	0	0
23	34.08	Dotriacontane	1.49	0.79	1.46	1.57	1.39	0	0.9	0.64	0.43	0.19	0	1.01
24	35.35	Elemol	0	0	0	0	0	0	0.14	0.3	0.23	0	0.32	0.21
		Total percentage	99.5	99.65	99.61	98.78	99.35	99.67	100	99.39	99.94	99.92	99.79	99.78

Santolina alcohol and p-menthane-3,8-diol,cis-1,3,trans-1,4 were two major compounds in the wild accessions and were not recorded in the regenerated genotypes. The occurrence of both compounds showed no preference to particular sites but santolina alcohol was higher in W1-5 compared to other sites (Table 4). On the other hand, 1-borneol, sabinyl acetate, and camphor are more common to regenerated genotypes but at lower proportions compared to the other major compounds, although 1-borneol was recorded at higher proportions in the regenerated plants of site 2 compared to other sites (Table 5). The proportion of these compounds varies between plants collected from different sites. Other four compounds, santolina triene, 2-methyl-2-octen-4-ol,4-, α-thujone, and ethyl cinnamate were represented in proportions ranging between 1% and 5% in the wild accessions. In total, the hydrocarbons constitute a small proportion of the oil while oxygenated constituents form the majority of the essential oil components.

### Diversity of wild accessions and regenerated genotypes based on essential oil constituents

**Fig. 7:**
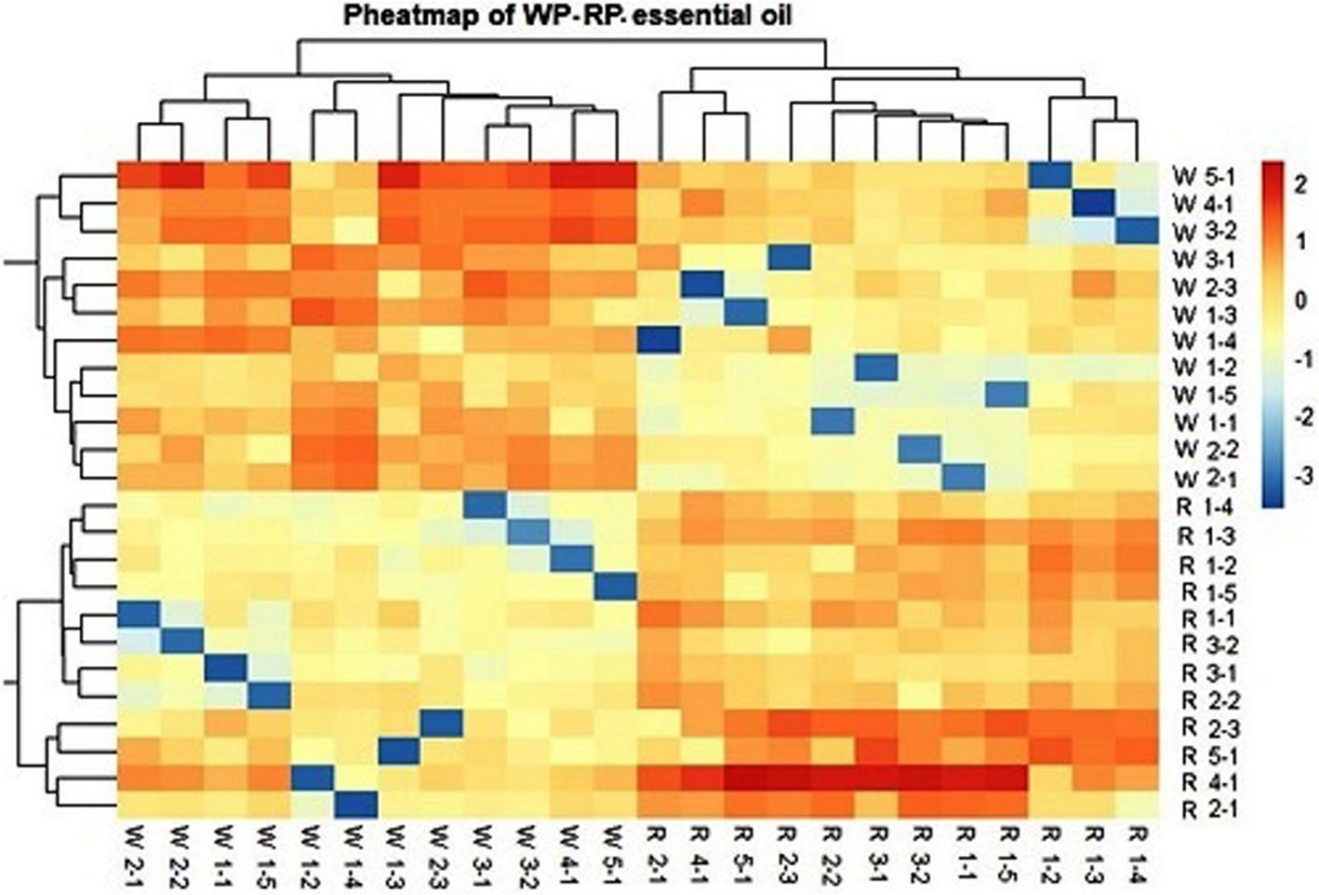
Heatmap illustrating the relatedness of the wild accessions and regenerated genotypes based on the variation of chemical constituents of the essential oil

The proportions of the chemical constituents of the essential oil were used to measure the relatedness of the wild accessions and regenerated genotypes using the pheatmap and ggplot2 packages to produce the heatmap illustrated in Fig. 7. In this heatmap, the scale of color is relative to the value of the divergence between accessions. A glimpse on the heatmap shows that the wild accessions and regenerated plants are distinguished as two separate groups. The high similarity among accessions of each group is indicated by the dominance of the red color in the part of the heatmap representing the similarity of the wild accessions to each other and the similarity of the regenerated genotypes to each other. On the other hand, some resemblance among wild accessions and regenerated genotypes is also indicated by the red color in most cells of the heatmap illustrating resemblance between wild accessions and regenerated genotypes (Fig. 7). Meanwhile, evident divergence was indicated by the blue color in 12 cells indicating divergence between every 12 wild accessions and 12 regenerated plants, However, no blue color was recorded for any wild accessions and their in vitro regenerated genotype indicating that the genotype of the wild accessions is more preserved in its regenerants than other in vitro regenerated plants.

### Correlation of essential oil constituents and morphological traits and ISSR fingerprinting

**Fig. 8:**
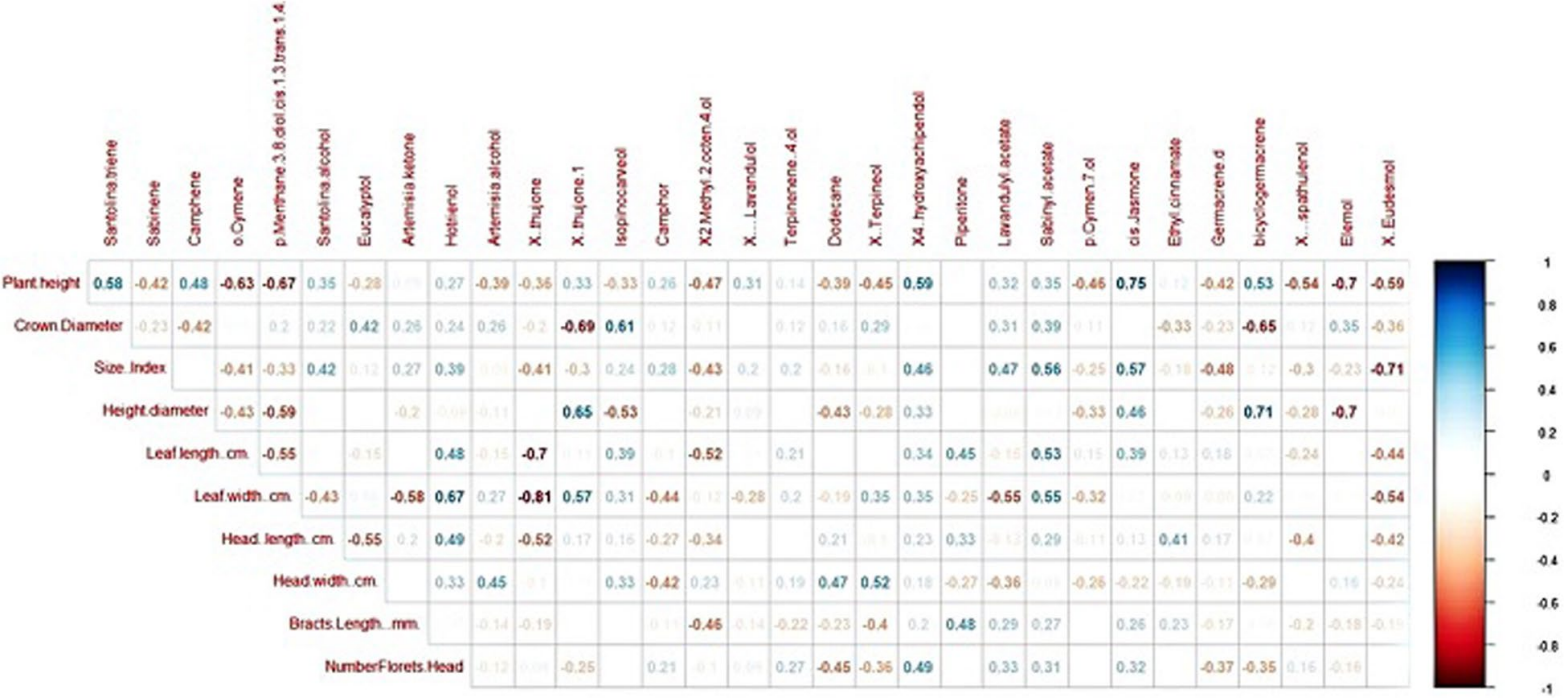
The correlation coefficients between the chemical components of the essential oils and the ten morphological traits for the 12 accessions of *A. fragrantissima* as two variables calculated by R-software

The correlation coefficients between the chemical components of the essential oil and the ten morphological traits for the 12 accessions of *A. fragrantissima*, as two variables calculated by R-software are shown in Fig. 8. The correlation coefficients are colored blue for positive correlations and colored red for negative correlations. In general, few morphological traits are correlated with particular compounds of the essential oil. Similarly, the Pearson correlation coefficient values between the ISSR fingerprinting markers and the volatile oil components in the wild accessions of *A. fragrantissima* are given in the supplementary Table 1A and the Pearson correlation coefficients of the ISSR markers and the volatile oil components, in the regenerated genotypes are given in the supplementary Table 1B. In general, few significant correlations were evident. For wild accessions, almost all monomorphic ISSR bands showed no correlation with oil components. As for regenerated genotypes, there is only one unique band that has a correlation with dodecane but piperitone showed correlation with 35 bands including 15 are +ve correlations and 20 −ve correlations. Artimisia ketone showed 14 correlations, including nine −ve and five +ve. Dodecane has 12 correlations, eight are -ve and four are +ve but only negative correlations were present in all genotypes derived from wild accession of site 1.

## Discussion

The foregoing data indicated that the analysis of ISSR fingerprinting revealed genetic variation between the wild accessions and their in vitro regenerated genotypes which represents limited genotypic fidelity of the in vitro propagated genotypes to their mother accessions. However, the differences between wild accessions were inherited to the regenerated genotypes as in vitro raised plants of the same site are closer to each other compared to genotypes regenerated from different sites. Some authors reported true-to-mother plant ISSR fingerprinting in the regenerated plants. Examples include *Rauwolfia tetraphylla*, [8], *Hedychium coronarium* [7], *Helianthus verticillatus* [9], and the blackberry [10]. However, genetic variations of plants in tissue cultures are well reported. Hamouda et al. [40] reported RAPD band pattern differences between mother plants and *in vitro* grown *Silybum marianum* genotypes indicating the existence of genetic variation. Meanwhile, Rady et al. [41] acknowledged inadequate genetic fidelity of in vitro raised *S. marianum* genotypes and Badr et al. [26] reported polymorphism in the *A. fragrantissima* plants. In the current study, the Euclidean distance UPGMA-CAP tree differentiated all wild accessions from the regenerated genotypes as two different groups. In each group, plants representing different sites were differentiated based on the analysis of ISSR polymorphism with the exceptions of the clusters from the two samples representing site 4 and site 5 respectively. However, in this tree, the regenerated genotypes are relatively more diverse compared to their wild mother accessions. The genetic divergence of the regenerated genotypes is clearly illustrated in the PCA scatter diagram. The genotypic differentiation of the examined wild accessions may be due to spatial and environmental differences in the sites from which the plants were collected [27], but the genetic variation between the in vitro raised genotypes may have been elicited by conditions and chemicals used for in vitro culture.

The GC-MS analysis of individual wild accessions of *A. fragrantissima* produced a wide 31 compounds in the volatile oil GC-MS chromatogram. In this respect, a comparative study of the volatile constituents of the essential oils of dried *A. fragrantissima* cultivated in Saudi Arabia and Egypt extracted using hydro-distillation and solid-phase microextraction (SPME) also revealed 31 compounds. Santolina alcohol, artemisia ketone, α-thujone, 4(10)-thujen-3-ol, β-thujone, were the predominant components in both extracts, with quantities varying with extraction method. The identified compounds include oil terpene hydrocarbons (aliphatic and cyclic) and their corresponding oxygenated isoprenoid derivatives and analogs. Most of the major volatile oil constituents identified in the present study were previously reported. Santolina alcohol (12.5–21.2%), artemisia ketone (13.2–23.8%), alpha-thujone (25.5–36.5%), constituted the main compounds for material from Sinai [42]. Santolina alcohol, artemisia alcohol, artemisia ketone, cis-thujone, and trans-thujone were also major constituents in the hydro-distilled oil and solvent extract of *A. fragrantissima* material from other parts in Egypt [28]. Artemisia ketone represented 49.53% and camphor 14.73% of essential oil from the leaves of *A. fragrantissima* growing wild in Yemen and the components of the essential were recommended for treatment of colon cancer [43]. Moreover, in the present study, some chemical compounds present in the wild accessions only and missing from the in vitro regenerated plants like santolina triene, sabinene, p-menthane-3,8-diol,cis-1,3,trans-1,4-, beta-thujone, isopinocarveol, 2-Methyl-2-octen-4-ol, β-eudesmol, and spathulenol, others present only in the in vitro plants such as limonene, 1-borneol, and dotriacontane. Moreover, piperitone was slightly reduced in regenerated plants derived from accessions of wild populations 1 and was only increased in the regenerated plants from W1-4 and W1-5. In the meantime, dodecane was generally increased. However, the values of the chemical compounds are given as a percentage and an increase of some compounds may be the result of a decrease in other compounds. The increase in the latter two compounds in the regenerated plants is congruent with their synthesis in the leaves of plants at the vegetative growth stage. At maturity, the vegetative leaves are often old and terminal branches and flowers are often the site in which volatile oil are synthesized. In vitro the plants were harvested before flowering and fruiting stage.

It has been reported that yield and chemical components in medicinal wild plants may be influenced by climate, soil, elevation, and topography, and their interaction [44–47]. In addition, the physiological situation of plants, time of collection, and different ecological conditions have a great effect on both the quantity and quality of essential metabolites in medicinal and aromatic plants in vitro and in vivo [48]. In *Silybum marianum*, Shokrpour et al. [17] showed that morphological traits, such as seed weight, flowering date, and plant height variation were determined by informative AFLP markers and qualitative and quantitative properties of the essential oil. In the *A. fragrantissima* unique bands were found associated with larger plant size and seed yield as well as better vigor [26]. In the current study, the environmental, soil, and other external factors prevailing in the study area have been minimized by a collection of wild accession of similar size and age at the same time. These precautions are important criteria for selecting accessions and sites for conservation and sustainable commercial use of *A. fragrantissima* as an important medicinal plant.

Gharib [49] found that the relative amounts of the major components of the essential oil of the *Pelargonium nervosum* regenerated shoots, were different from those present in the partially differentiated callus and parent plant and only citral was the main oil components present in all cultures [49]. The GC-MS chromatogram of essential oils of dried aerial parts of *Salvia sclarea* L. plants, regenerated in vitro and reproduced from seeds, were similar, although the yield of essential oil from in vitro plants was lower than the oil yield isolated from *in vivo S. sclarea* plants [50]. In *Teucrium scorodonia* L. ssp. scorodonia, the components of the essential oil from the micropropagated plants and the seed-derived plants were qualitatively similar but, quantitative differences were evident [51]. In the current study, acclimatized in vitro gown regenerants of the same mother plants were of similar size and age and grown under the same conditions. In the regenerated genotypes of *A. fragrantissima*, a total of 24 volatile compounds were detected while in the wild accessions, 31 compounds were detected in the GC-MS chromatogram. This variation is due to is the fact that mature wild plants and the in vitro propagated plants were harvested at different developmental. Although largely species specific, volatile oil components amount, and types vary with the age of the plant and the environmental condition. Future optimization of growth conditions like temperature or culture age and elicitors additions would help achieve the best in vitro conditions for metabolite production. Four major compounds are common to both wild and regenerated plants, these are artemisia ketone, alpha-thujone, dodecane, piperitone. DNA fingerprinting and essential oil content variation was also common in callus-derived regenerants of *Curcuma longa* and was attributed to soma-clonal variations in derived regenerants [52]. In the in vitro grown genotypes, chemotype-specific markers are less likely reported in *Silybum marianum* by Shokrpour et al. [17] and in *Thymus vulgaris* by György et al. [18]. The latter authors assumed that no chemotype-specific markers can be found without sequence information. Under plant morphogenesis, DNA methylation and histone modifications are very susceptible to those in vitro environmental conditions which may induce genetic and epigenetic variability in the regenerated plants [53].

## Conclusions

The genetic diversity analyses using clustering and principal component analyses generally differentiated the mother wild accessions of *A. fragrantissima* from their regenerated genotypes. The resemblance of wild accessions from different sites and the regenerated genotypes indicates that genetic variation of wild accessions was inherited to their *in vitro* propagated genotypes. The number of volatile oil compounds in the wild *A. fragrantissima* accessions was 31 compounds while in the in vitro propagated plants only 24 compounds were detected. The amount of volatile oil is much greater in the wild accessions compared to regenerated plants. Four major compounds are common to both wild and regenerated plants, but considerable variation in the types and amounts of compounds was evident among the wild accessions and their in vitro regenerated genotypes. In vitro conditions elicited higher genetic variation that may be associated with diversity in essential oil components, but no particular correlations are persistent between ISSR markers and volatile oil components.

## Supplementary Information

**Additional file 1.**

## Data Availability

Not applicable.

## Abbreviations

ISSR: Inter simple sequence repeats
SCoT: Start codon targeted polymorphism
RAPD: Random amplified polymorphic DNA
SRAP: Sequence-related amplified polymorphism
GC-MS: Gas chromatography-mass spectrometry
PCR: Polymerase chain reaction
MS medium: Murashige and Skoog medium
BA: Benzyl adenine
NAA: 1-Naphthaleneacetic acid
CAP: Community Analysis Package-5
The PAST: Paleontological Statistics
UPGMA: Unweighted Pair Group Method with Arithmetic Mean
PCA: Principal coordinates analysis
pr: Primer
W: Wild accessions
R: Regenerated genotypes
